# Metal-Oxide Based Nanomaterials: Synthesis, Characterization and Their Applications in Electrical and Electrochemical Sensors

**DOI:** 10.3390/s21072494

**Published:** 2021-04-03

**Authors:** Enza Fazio, Salvatore Spadaro, Carmelo Corsaro, Giulia Neri, Salvatore Gianluca Leonardi, Fortunato Neri, Nehru Lavanya, Chinnathambi Sekar, Nicola Donato, Giovanni Neri

**Affiliations:** 1 Department of Mathematical and Computational Sciences, Physics Science and Earth Science, University of Messina, Viale F. Stagno D’Alcontres 31, I-98166 Messina, Italy; salvatore.spadaro@unime.it (S.S.); fortunato.neri@unime.it (F.N.); 2 Department of Chemical, Biological, Pharmaceutical and Environmental Sciences, University of Messina, Viale F. Stagno D’Alcontres 31, I-98166 Messina, Italy; giulia.neri@unime.it; 3 Institute of Advanced Technologies for Energy (ITAE)—CNR, Salita Santa Lucia Sopra Contesse 5, I-98126 Messina, Italy; leonardi@itae.cnr.it; 4 Department of Bioelectronics and Biosensors, Alagappa University, Karaikudi 630003, India; lavan153@gmail.com (N.L.); sekar2025@alagappauniversity.ac.in (C.S.); 5 Department of Engineering, Messina University, I-98166 Messina, Italy; ndonato@unime.it (N.D.); giovanni.neri@unime.it (G.N.)

**Keywords:** metal-oxide, nanohybrid, conductometric sensors, gas sensing, electrochemical sensors, biosensing

## Abstract

Pure, mixed and doped metal oxides (MOX) have attracted great interest for the development of electrical and electrochemical sensors since they are cheaper, faster, easier to operate and capable of online analysis and real-time identification. This review focuses on highly sensitive chemoresistive type sensors based on doped-SnO_2_, RhO, ZnO-Ca, Sm_x_-CoFe_2−x_O_4_ semiconductors used to detect toxic gases (H_2_, CO, NO_2_) and volatile organic compounds (VOCs) (e.g., acetone, ethanol) in monitoring of gaseous markers in the breath of patients with specific pathologies and for environmental pollution control. Interesting results about the monitoring of biochemical substances as dopamine, epinephrine, serotonin and glucose have been also reported using electrochemical sensors based on hybrid MOX nanocomposite modified glassy carbon and screen-printed carbon electrodes. The fundamental sensing mechanisms and commercial limitations of the MOX-based electrical and electrochemical sensors are discussed providing research directions to bridge the existing gap between new sensing concepts and real-world analytical applications.

## 1. Introduction

**Figure 1:**
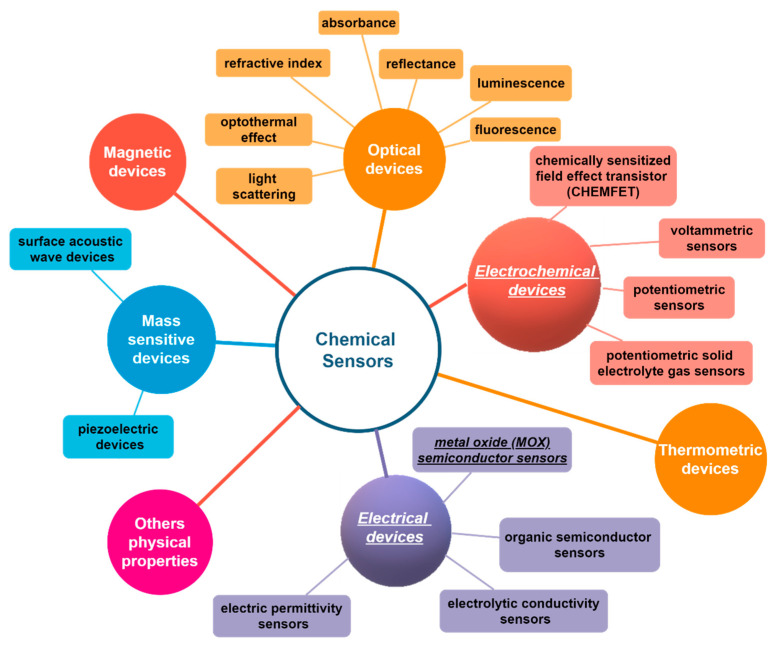
Scheme of the chemical sensors classification from International Union of Pure and Applied Chemistry (IUPAC) [1].

Today, chemical sensors represent a class of devices of outmost importance both from the scientific and applicative point of view. Since 1991 the International Union of Pure and Applied Chemistry (IUPAC) provided the definitions and classification of chemical sensors: “A chemical sensor is a device that transforms chemical information, ranging from the concentration of a specific sample component to total composition analysis, into an analytically useful signal” [1]. Figure 1 reports a scheme with the classification adopted by IUPAC.

Among the typology of chemical sensors, electrical and electrochemical ones are the most simple and therefore have been largely investigated and utilized in practical applications: either gases and substances in liquid phase can be detected and quantified by simple electrical and electrochemical transduction platforms. These devices have now reached a large market volume: the Electrochemical Sensor Market was valued at USD 6.19 billion in 2020 and is expected to reach USD 11.83 billion by 2026 [2].

The popularity of sensor devices to detect gases is due to the increased development of solid-state sensors, the miniaturization of gas detection devices and the micro electro-mechanical system (MEMS) technology advent. Indeed, compared with conventional analytical instruments such as gas chromatography (GC) and high-performance liquid chromatography (HPLC), chemical sensors are less costly, easier to operate and capable of online real-time identification.

Apart from the carbon based and metal nanomaterials, metal oxides (MOX) attracted great attention, due to their chemical characteristics and functional properties [3,4,5], for the development of chemical sensors [6,7] applied to achieve a safer working environment. In compliance with government regulations such as Control of Substances Hazardous to Health (COSHH) and Occupational Safety and Health Administration (OSHA) regulations, MOX based sensors have been recently adopted to efficiently reveal the presence of toxic and combustible gases (i.e., hydrogen sulfide, carbon monoxide) to withstand high humidity and temperature. All of that to avoid explosions in manufacturing and chemical industries. Moreover, MOX based nanomaterials chemical sensors are increasingly used in the automotive field to optimize cabin air quality, as fuel emission detectors and as fast point-of-care testing and monitoring devices in biomedical field. For example, very recently, a new handheld gas sensor for Airborne SARS-CoV-2 virus diagnosis from exhaled breath was awarded an NSF RAPID grant, demonstrating the potentiality of these devices in the biomedical field.

Among MOX, metal oxide semiconductors (MOS) with sizes in the range of 1–100 nm have been considered as promising candidates for gas detection by means of electrical transduction platforms [8]. The processes involved in gas sensing with MOS gas sensors are extremely complex and their understanding, which is needed for developing smart sensors, demands promising investigation techniques that must be applied in operation conditions. These are the main issues that are still open today. Parameters like sensitivity, selectivity, response time and stability of gas sensors can be further improved by the addition of different dopants, which act to change the activation energy, to generate oxygen vacancy or to change the electronic structure/band gap [9]. The doping of nanowires, nanotubes, core-shell nanostructures and nanofibers is paving the way for newer and better gas sensor materials [10,11]. More recently, MOX have been receiving great attention mainly in the field of biosensors due to their high potential and versality to become very competitive materials for modifying the morphology, chemical stability and physicochemical interfacial properties of conventional sensing materials [12]. In fact, MOX can be assembled to form tandem heterostructures [13], hybrid structures [14] or composite structures [15] with advanced electrochemical properties which can be adapted for a specific biosensor application.

MOX materials find large applications also in the fabrication of biosensors. Such devices consist of sensitive biometric elements, transducers and signal analysis systems allowing the rapid detection of various trace-level analytes [16]. The sensitive element selectively reacts (enzyme) or binds (antibody) with analytes. Therefore, the transducer captures the results of the interaction between sensitive material and analyte. These could be changes in number of transferred electrons in the case of redox enzymes or changes in mass or potential for various sensors based on immunosensing principles. However, for their biosensing applications there are several issues to overcome such as organic/inorganic interface compatibility, increasing the carrier charge mobility, while decreasing the electron-hole recombinations. Furthermore, for an effective commercialization, an important prospect is the prolongation of lifetime of the sensors, as well as the stability and reliability of the sensor signal, especially in humid conditions. Thus, a great deal of researchers’ attention will be focused on novel inorganic nanomaterials.

Besides the biosensor field discussed above, MOX thin film transistor (TFTs) could contribute to environmental sensing and automation biosystems [17]. However, this field is almost completely unexplored. The chemically modified biosensors can be regarded as an efficient technology for the determination of various biomolecules.

A lot of literature has been produced through the years covering all issues regarding metal, carbon and MOX materials, such as their preparation and characterization, sensing mechanism and applications. To complete this, the core objective of our review is to provide the most complete and exhaustive picture on the types of sensors available today in the market, highlighting their advantages and disadvantages in the specific field in which they are used, focusing the attention on the sensing performances of novel MOX based sensors developed in our joint laboratories at the University of Messina (Italy) and University of Alagappa (India). In particular, we highlight the enhanced sensing properties achieved by using MOX-based modified electrical and electrochemical sensors illustrating the corresponding sensing mechanisms. For example, we show improved ammonia sensing response of V-doped ZnO:Ca nanopowders prepared by sol–gel synthesis (see Section 3). Additionally, we discuss about the properties of MOX-modified glass carbon and scree-printed, carbon-based electrochemical sensors with respect to the electrocatalytic characteristics collected using the common carbon electrode materials. In such a way, we shed light on the advantage of the simultaneous detection of different analytes with similar oxidation potential such as epinephrine, uric acid and ascorbic acid (see Section 5). Therefore, this review will serve as a source of knowledge for the future development of innovative and more performing MOX and specifically MOS based sensors for quantitatively and selectively measuring target species in complex systems. To this purpose, specific applications of different electrical and electrochemical sensors based on MOX nanocomposites in real and analytical situations will be discussed together with their limitations. We believe that this review may help to provide research directions by specifying existing hindrances and can also aid in designing novel materials.

## 2. Conductometric Type Sensors: Building Basics and Sensing Mechanisms

MOS-based conductometric gas sensors are the most used and studied electrical devices designed for the control of toxic and inflammable gases in technological processes and surrounding atmosphere [18]. Conductometric sensors have a simple structure which consists of two elements, a sensitive conducting layer and contact electrodes. To make the measurement, a DC voltage usually in the range of 1–10 V is applied to the device and the current flowing through the electrodes is monitored as the response. The sensing material bridges the gap between two electrodes or coats a set of interdigitated electrodes, printed on an insulating ceramic, a plastic flexible foil or a silicon substrate [19]. For those sensors which do not work at room temperature, a heating micro-resistance can be included on the bottom side of the sensor to bring the sensitive material to the optimal working temperature.

**Figure 2:**
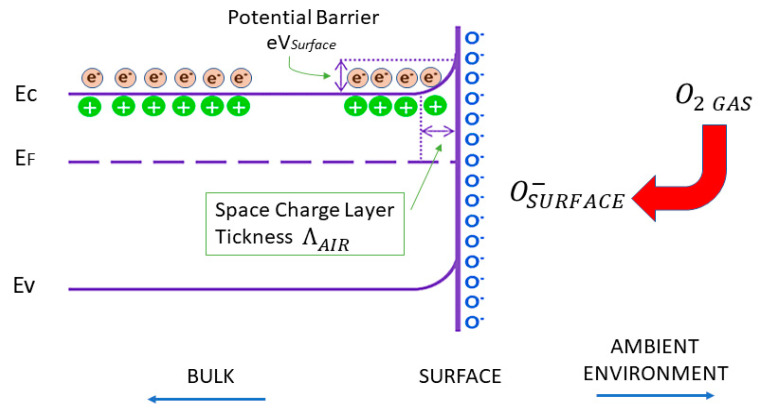
Schematic diagram of band bending after chemisorption of charged species (here the ionosorption of oxygen). E_C_, E_V_, E_F_ and eV_surface_ denote the energy of the conduction band, valence band, the Fermi level and the potential barrier, respectively while Λ_air_ denotes the thickness of the space-charge layer. The conducting electrons are represented by e^–^ and the symbol + represents the donor sites.

The basis of the operation of conductometric sensors is the change in resistance/conductivity of a sensitive layer under the effect of reactions (adsorption, chemical reactions, diffusion, catalysis) taking place on the surface of the sensing layer. The chemical species interact with the sensitive layer and thus modulate its electrical conductivity (essentially trapping of electrons at adsorbed molecules and band bending induced by these charged molecules are responsible for a change in conductivity). The most accepted mechanism, explaining the sensitivity of n-type MOX-based sensors, includes the role played by the chemisorbed oxygen [20]. The negative charge trapped in these oxygen species causes an upward band bending and thus a reduced conductivity compared to the flat band situation. As shown in Figure 2, when O_2_ molecules are adsorbed on the surface of MOX, they would extract electrons from the conduction band E_c_ and trap the electrons at the surface in the form of ions. This will lead a band bending and an electron depleted region, called space-charge layer, whose thickness coincides with the length of the band bending region. Reaction of these oxygen species with reducing gases or a competitive adsorption and replacement of the adsorbed oxygen by other molecules decreases and can reverse the band bending, resulting in an increased conductivity [20]. O^−^ is believed to be dominant at the operating temperature of 300–450 °C which is the work temperature for most metal oxide gas sensors. These changes in the film conductivity are thus correlated to the concentration of the chemical species.

The main advantages of MOX conductometric sensors are: (1) ease of fabrication using thin and thick film technologies, (2) simple operation and (3) low production cost. Specifically, reversibility, rapid response, longevity and robustness are other merits of metal oxide gas sensors. However, conductometric MOX sensors are not highly selective, and much effort has been involved in devising materials and methods of operation to improve specificity [21,22]. During exposure, gases interact with the sensing material producing a modulation of the resistance value which represents the response of the sensor. Hence, by measuring the increase or decrease of electrical resistance (or conductivity), the type (oxidizing or reducing) and concentration of gas (i.e., H_2_, CO, NO_2_) or Volatile Organic Compound, VOC, (i.e., acetone, ethanol) can be estimated [23]. Easy measurement of the electrical properties with only two electrodes is a key factor in their preference and supplying safety [24,25].

Many sensing materials can be employed as active layer in these devices including MOS, graphene, carbon nanotubes and metal nanoparticles in self assembled monolayers or conductive polymers [26,27,28,29,30]. Chemical composition and structural properties are the main factors affecting MOS chemoresistive sensor response toward gases and their stability over time [31,32]. On the other hand, different sensing properties have been obtained changing materials morphology, mainly remarking nano-scale peculiarities to improve gas sensors [29,33]. The doping of MOX materials or any approach used to “create oxygen defects” results in a large concentration of carriers, mobility and change in electrical resistivity. In particular, doping with metallic ions (Al, Fe, Co, Cu, Ag, etc.) is an effective method for enhancing sensing capability of about two orders of magnitude with respect to the undoped samples [33]. The substituted atoms can act as reactive sites for gas adsorption [34] and can cause extrinsic electronic states [35,36].

Noble metal species with high-effective oxidation catalytic activity can be used to enhance the sensitivity of pure MOX due to the “spillover effect” [37]. Moreover, good catalyst supporting materials are also a key point to determine how much potential of catalysts can be developed. So, the structure of MOX layers is very important. High surface areas are necessary to obtain highly-dispersed catalyst particles. Furthermore, high surface areas can provide large reaction contact area between gas sensing materials and target gases. Therefore, porous structures with high surface areas seem to be the standard structure of MOX gas sensor layers, while one-dimension materials are prospective material platform for the next generation of durable conductometric gas sensors due to open surface, high gas sensitivity and long-term stability.

**Figure 3:**
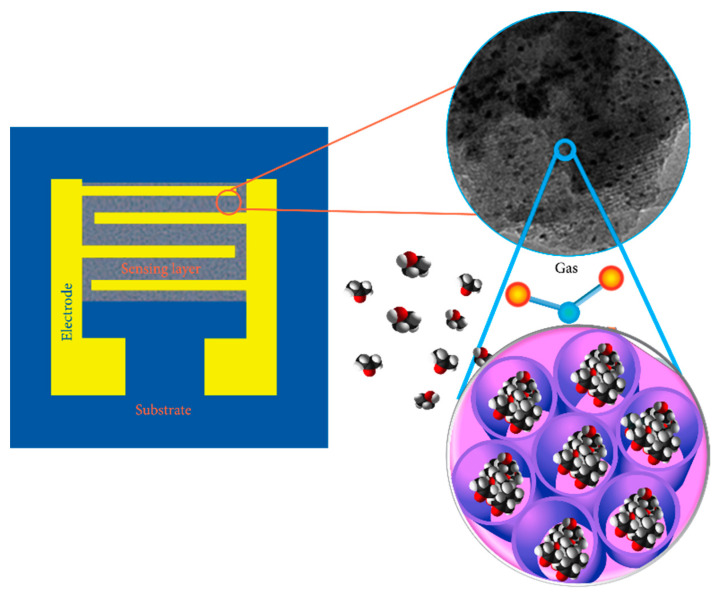
Schematic design of a gas sensor based on meso-nanoporous metal oxides. The device takes advantage from the porous structure and large specific surface area of the material to enhance the sensing performances. Reprinted under the terms of the Creative Commons Attribution License from Ref. [44].

Particularly interesting are the nanoporous and two-three dimensional structures of MOX such as nanowires, nanorods and nanotetrapods [38,39,40,41,42]. Nanostructures have an extremely high surface/volume ratio and, since the sensitive part of the oxide is their surface which comes into contact with gases and others volatile compounds in air [43]. It is easy to understand how this property can greatly influence the two main processes involved, when the MOX surface reacts with the surrounding atmosphere containing oxygen and target gases (see Figure 3).

The mechanism of gas sensing on MOS sensors has been largely investigated in the last several decades [45]. It is assumed that the first process is the diffusion of the analyte gas from the atmosphere toward the oxide semiconductor surface. The diffusion process is improved if the sensing film has a micro-, meso- or nanoporous structure [43,44]. The second process consists in the charge-transferring interaction between the analyte gas and the oxide surface. This mechanism depends on the gas adsorption, the change of charge carrier concentration in proximity of the oxide surface, and by the surface reactions [46,47,48]. Particularly, nanostructured sensitive layers allow complete electron depletion and effective gas diffusion, thus yielding high sensing performance in terms of short recovery times and low detection limits [49,50]. Finally, the sensor working temperature plays an important role either in the formation of reactive species and chemisorbed reactive oxygen species (ions) [51,52], according to the following reactions:

$$O_{2\left(ads\right)}+e^{-}⇆O_{2\left(ads\right)}^{-}\;(<100\;{}^{\circ}C)$$

$$O_{2\left(ads\right)}^{-}+e^{-}⇆2O_{\left(ads\right)}^{-}\;(100–300\;{}^{\circ}C)$$

The formation of oxygen ions results in the capture of electrons from conduction band of the surface layer, determining an alteration in conductivity of the MOS [52]. The increase or decrease in conductivity depends on the type of majority carriers in the semiconducting metal oxide material (n-type or p-type) and on the nature of the probed gas molecules (oxidizing or reducing). In the case of n-type nanostructured oxides, the electrons are “removed” from the conduction band of the surface layer thanks to the action of the adsorbed oxygen molecules. In this way, negatively charged chemisorbed ions will be formed. In particular, at room temperature, surface oxygen ions will be $O_{2}^{-}$ type [51]. Consequently, we have the formation on the oxide surface of a depletion zone and a potential barrier, which produce a decrease in conductivity or, analogously, an increase in the resistance of the oxide layer, due to the loss of electrons [53].

In each case, the analyte gas plays an important role in the detection mechanism. Indeed, in the case of reducing gas (donor), i.e., NH_3_, H_2_, H_2_S, HCHO etc., the chemical reaction, taking place on the oxide surface, releases electrons which are reintroduced into the depletion layer. It follows a lowering of the potential barrier level and, therefore, an increase in conductivity (i.e., decrease in resistance). On the other hand, when the target gas is an oxidant (acceptor), such as O_3_, NO, CH_3_COCH_3_, Cl_2_, NO_2_, etc., the reaction with chemisorbed oxygen ions causes a further loss of electrons from the depletion layer, widening it and producing an increase in the potential barrier. The process described is responsible of a decrease in conductivity, or equivalently, an increase in the resistance of the oxide layer.

**Figure 4:**
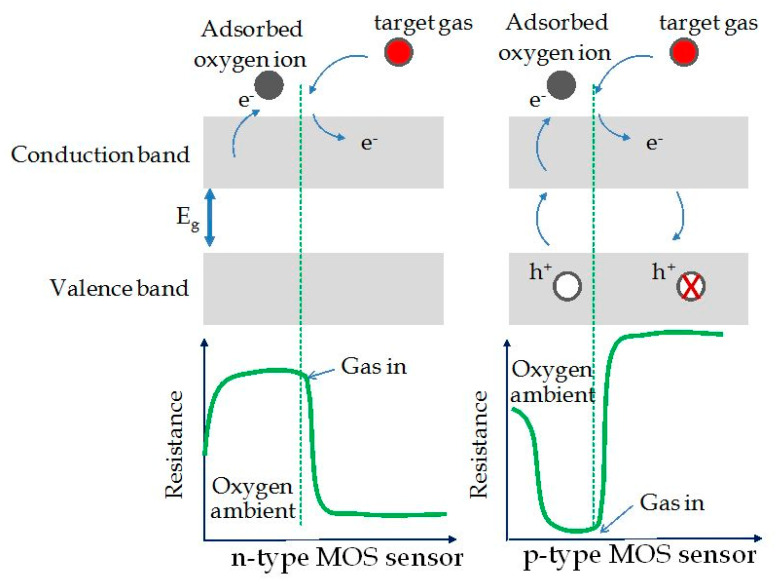
Schematic diagram for change of the sensor resistance upon exposure to the target gas (reducing gas) in the cases of n-type and p-type metal oxide sensors. Reprinted under the terms of the Creative Commons Attribution License (CC BY 3.0) from Ref. [54].

Figure 4 shows a schematic diagram of sensor resistance changes upon exposure to the target gas (reducing gas) in the cases of n-type and p-type MOX sensors, respectively.

If the metal oxide is n-type, a lowering in resistance will take place if exposed to reducing gas; on the other hand, the material shows an increase in resistance if exposed to oxidizing gas [45,55]. In the case of p-type nanostructured oxides, the gas detection mechanism is always linked to the change in resistance of the oxide layer, following the oxidation or reduction reactions that occur between its surface and the target gas. In this case, the key factor is the change in the concentration of p-type carriers. At room temperature, when the air-analyte gas mixture interacts with the oxide surface, two processes take place: (1) the formation of oxygen ions $O_{2}^{-}$ due to the molecules that have been adsorbed, and (2) the capture of electrons from the oxide conduction band. In this way, there will be an increase in p-type carriers concentration on the surface layer, with a lowering of the Fermi level, or an increase of the oxide conductivity (i.e., decrease of resistance) [56,57]. Specifically, if the sensor is exposed to reducing gases, whose molecules are adsorbed on the oxide surface, the electrons released will recombine with holes, causing an increase of the Fermi level and a reduction in the p-type carriers concentration (reaction between gas and $O_{2}^{-}$ ions). The result is a decrease in conductivity or equivalently an increase in resistance. Otherwise, once the sensor is exposed to oxidizing gases, whose molecules are adsorbed by the metal oxide surface, electrons were captured, forming negatively charged chemisorbed oxygen ions. This mechanism leads to an increase in the concentration of holes (majority carriers), which results in an increase of conductivity and, therefore, a decrease in resistance of the sensitive layer. To summarize, for p-type MOX, an increase in resistance if exposed to reducing gas, while a decrease in resistance if exposed to oxidizing gas were observed. This is exactly the opposite behavior with respect to n-type MOX sensing materials [54,58,59].

Thus, in metal-oxide and metal-doped oxide nanostructured sensing layers, a well- defined control of film growth process is essential to avoid agglomerative formations and unexpected ion positions in the crystal structure, in turn, to limit the decrease of gas adsorption process. For what has been said: “a careful engineering control over the metal oxide structure and sensor design is mandatory to obtain high stability as well as high gas sensitivity for devices” [60,61]. Besides the internal causes limiting metal oxide sensors mentioned above, external causes, such as temperature and humidity, also play an important role. Humidity decreases the sensitivity so preventing measurements reproducibility. Fortunately, it can be eliminated by heating to high temperatures (usually >400 °C) [20]. Nevertheless, among all the types of sensors nowadays available, the chemiresistive ones are distinguished by low cost in terms of production and operation, long lifetime, good stability and reproducibility of measurements, as well as their high response speed and sensitivity because they allow the quantitative estimation of target gas concentration variation by a direct measurement of electrical resistance [62,63]. Additionally, being usually small and low in power consumption, it is very simple to integrate them into distributed sensor networks or in everyday objects to turn them into smart objects, in view of the expansion of the emerging Internet of Things (IoT) experience.

## 3. An Overview on MOX Nanomaterials Used for Gas Sensing

Recently, there was an increased interest towards the applications of gas sensors based on semiconductor MOX. For instance, TiO_2_, SnO_2_ and ZnO have been successfully applied for the detection of combustible and toxic gases, principally for monitoring the environmental pollution and to secure the home/industrial ambient [20,64,65]. In details, for the sensing of hydrocarbons, oxygen, CO, H_2_ and NO_2_, devices based on ZnO have been adopted in a real-time fashion, with a particular enhancement for doped nanostructures by Al, Ga, In and Sn [66].

From our side, some of us investigated the sensing properties of V-doped ZnO:Ca nanopowders that were synthetized by the sol–gel technique. The results showed an increase in the resistive sensor response for the detection of ammonia (NH_3_), ascribed to the combined impacts of V, ZnO and Ca. This has very important fallouts concerning gas sensing for environmental detection, automotive-chemical industry and for medical purposes. In fact, just revealing 1 ppm of ammonia is particularly important both in environmental pollution and biomedical applications. Note that the ammonia limits are fixed to 35 and 25 ppm for short term and long term exposure, respectively [67].

**Figure 5:**
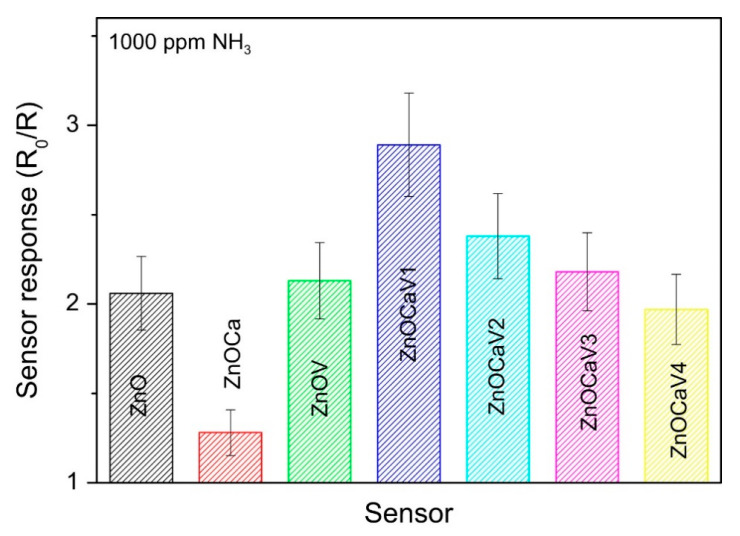
Detection response of 1000 ppm of ammonia for the investigated sensors. Reprinted from Journal of Solid State Chemistry, Vol 226, Fazio E. et al., Ammonia sensing properties of V-doped ZnO:Ca nanopowders prepared by sol–gel synthesis, Pages No. 192–200, Copyright (2015), with permission from Elsevier [67].

Figure 5 reports the detection response of 1000 ppm of ammonia for the investigated sensors, working in air under the same conditions. As it can be observed, sensors with V components display the most enhanced response on average, whereas the binary ZnO:Ca has the weakest response for ammonia detection. Therefore, it seems that the inclusion of vanadium is responsible of this enhancement although only if combined with calcium. In fact, the ZnOV sample shows a response that is nearly the same than that of the reference ZnO sample. Furthermore, the response for ZnO:CaV_x_ sensors depends on the V amount, being the highest for the ZnO:CaV_1_ sample. In particular, this last sensor has a sensitivity toward the ammonia detection which is about 2.85 × 10^−3^ ppm^−1^. For this specific purpose, this sensitivity value is higher than that shown by carbon nanotubes based sensors that are also less cheap [68].

CO_2_ species are considered the main responsible of the greenhouse effect and global warming [69]. The concentration range of CO_2_ is between 0.03% (300 ppm), corresponding to the CO_2_ concentration present in uncontaminated atmospheric air, and 0.3% (3000 ppm), typically found in closed and highly populated ambient. CO_2_ is mainly monitored by infrared (IR)-based gas sensors, but MOX materials showing sensitivity to CO_2_ have also been recently exploited for developing conductometric sensors for monitoring this gas. For example, many efforts in our laboratory and from other research groups have been made to optimize CO_2_ sensors based on ZnO sensing elements [70,71], which incorporated suitable dopants (i.e., Al). However, a precise control of particle shape and size, as well as of the amount of dopants, is considered essential to ensure high sensing performance, without dramatically alter electrical characteristics and reactivity of ZnO, when interacting with gaseous species [72].

Dhahri et al. investigated the performance of a resistive CO_2_ sensor based on ZnO:Ca nanoparticles, synthesized by sol-gel method [73]. The improvement of the Ca-doped ZnO sensor response (S = (ΔR/R_0_) × 100 = 113 to 5% CO_2_) with respect to the ZnO sensor, was observed in terms of the higher adsorption of CO_2_ on the semiconductor surface in the presence of Ca dopant. In fact, the presence of Ca promotes the formation of carbonates species as confirmed by the trend of the intensity of IR band at 1420 cm^−1^ (due to the formation of carbonates species), versus Ca loading and also by the response of the sensors versus the intensity of IR band at 1420 cm^−1^ (see Figure 9 in Ref. [73]). On the overall, the substitutional doping of ZnO, with lower or higher valence (e.g., I or III group) impurities, is known to result in enhanced carrier concentration and lowered resistivity (p- or n-type doping) [74]. On the contrary, the incorporation in the lattice of isovalent ions (e.g., Ca^2+^), having larger ionic radius with respect to Zn^2+^ ions, creates large lattice distortion leading to an increase in the adsorption of acidic CO_2_. This is due to dopant-induced modification of the acid-base properties of the ZnO surface [75], finally resulting in improved sensor response.

**Figure 6:**
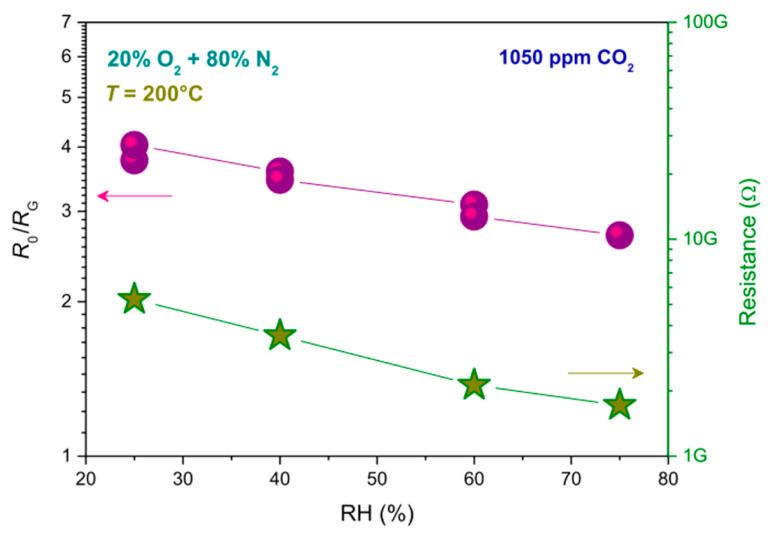
Relative humidity (RH) effect on baseline resistance (right *y*-axis) and response (R_0_/R_G_, left *y*-axis) of nanofibers-based sensors. Reprinted with permission from Pantò, F. et al., CO_2_ sensing properties of electro-spun Ca-doped ZnO fibers. Nanotechnology 2018, 29, 305501 [76].

Gas sensing properties are strongly affected by nanomaterials properties, such as morphology and composition. Here, we report the gas sensing response of Ca-doped ZnO nanofibers at different Ca to Zn loading ratio (1:40 or 1:20) [76]. Generally, electro-spun fibers are characterized by a very large surface area and high porosity, unique properties to enhance sensor performance for the detection of CO_2_. The ZnO:Ca fibers, produced by Pantò et al. [76], are constituted by interconnected grains of oxide with the hexagonal wurtzite structure of zincite. The efficient sensor response is given by the combined effect of the fiber morphology and the presence of Ca-ion sites. In fact, the first effect favors diffusion processes inside the sensing layer by the gas molecules, whereas the second favors CO_2_ adsorption. However, the sensing response reduces on increasing the relative humidity (RH) because of the competing absorption between water and oxygen molecules on the sensor surface. This phenomenon has to be investigated especially for the evaluation of air quality. Figure 6 reports the baseline resistance (right *y*-axis) and the response to CO_2_ of nanofibers-based sensor (left *y*-axis), in terms of the ratio between the sensor resistance in air (R_0_) and in the presence of the gas (R_G_), by varying RH from 25% to 75%. A decrease of both these parameters can be easily seen on increasing RH.

It is well known that replacing hydrocarbons with hydrogen as energy source has many advantages. However, hydrogen shows high flammability and is an odourless gas [77,78]. Indeed, its detection is fundamental within all its development chain, from production to use. Mass spectrometry and gas chromatography cannot be used for H_2_ detection due to their large dimension and cost. Hence, most reliable sensors are being developed to accomplish the industrial and safety needs. One example is constituted by MOX based conductometric sensors although they show a scarce long-term stability especially when working at high hydrogen concentration and/or at high temperature. This is caused by the reducing effect of hydrogen so not allowing, at the moment, the employment of these kinds of sensors for practical applications [79].

**Figure 7:**
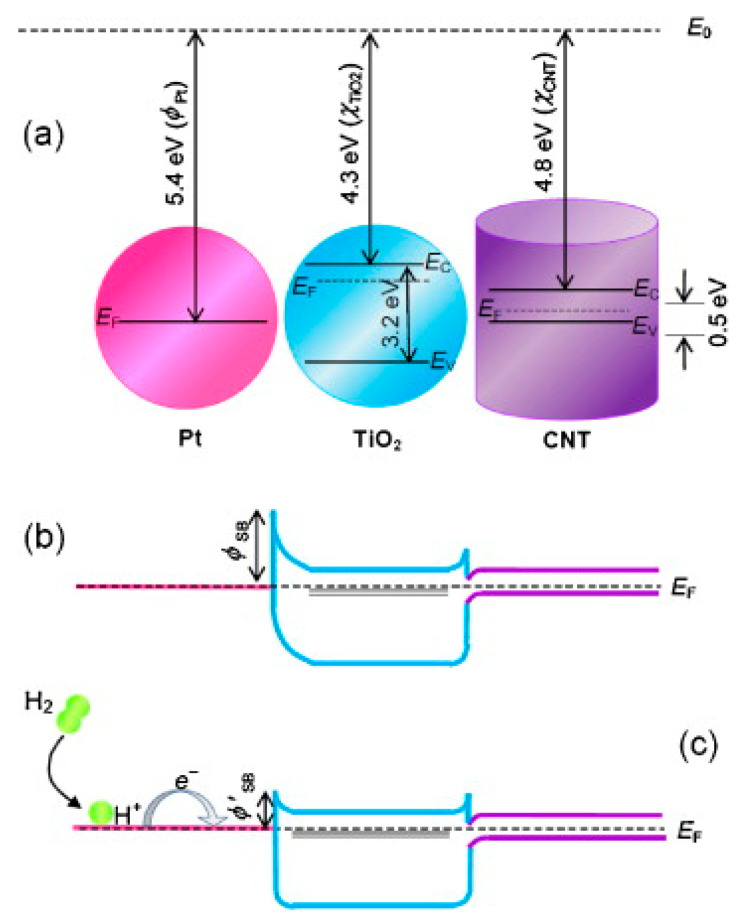
Schematic band diagrams for (**a**) Pt, TiO_2_ (large gap n-type semiconductor) and CNTs (p-type semiconductor), and (**b**) Pt/TiO_2_/CNTs nanocomposites. (**c**) Drawing for the charge transfer occurring after H_2_ dissociation and characterizing the sensing mechanism. Reprinted from Sensors and Actuators B: Chemical, Vol 178, Santangelo S. et al., On the hydrogen sensing mechanism of Pt/TiO_2_/CNTs based devices, Pages No. 473–484, Copyright (2013), with permission from Elsevier [81].

Moreover, resistive sensor devices have been tested for H_2_ sensing. Recently, a resistive sensor, based on carbon nanotubes (CNTs), that utilizes 2 wt% Pt/TiO_2_/CNTs as an active material, has been proposed by De Luca et al. [80] for monitoring H_2_ in inert atmosphere. Interestingly, results of this study have shown that the 2 wt% Pt/TiO_2_/CNTs-based sensor operates at near-room temperature (NRT) and responses to a very wide range of H_2_ concentrations (5–100%) while, under the same conditions, devices based on 2 wt% Pt/CNTs and 2 wt% Pt/TiO_2_ exhibit much lower responsiveness. The sensor working mechanism is ascribed to the quenching of the carbon nanotube conductance, following the oxide mediated electron transfer from the metal. The differences in stoichiometry, agglomeration degree of the nanosized TiO_2_ phase and surface contact with CNTs (introduced by the variation of the CNT load) account for the changes in responsiveness of the sensors based on Pt/TiO_2_/CNTs composite [81]. Santangelo et al. [81] interpreted their results in terms of the formation of a Schottky barrier at the Pt/oxide interface provoked by the electron transfer from TiO_2_ to Pt because of the work function of platinum (ø_Pt_ = 5.4 eV) is higher than the electron affinity (E_ea_) of TiO_2_ (E_ea_ = 4.3 eV) as indicated in Figure 7a. At the same time, at the oxide/CNTs interface a n-/p-type hetero-junction can form with a corresponding band gap of 0.5 eV, given that E_ea_ (CNTs) = 4.8 eV. Therefore, once hydrogen molecules were adsorbed and activated onto Pt nanoclusters, can dissociate and diffuse, throughout a “spill-over” mechanism [82], within the metallic system so reducing the work function [81] (Figure 7b,c).

Indeed, hydrogen species behave as surface donors by transferring an electron to the conduction band of titania and then to the graphitic network by taking advantage of the edges position corresponding to the conduction bands of TiO_2_ and CNTs. This mechanism, which is involved also for photo-injected carriers in TiO_2_/CNTs catalysts [83], provokes the decrease in the concentration of H^+^ species within CNTs and the subsequent enhancement of the electrical resistance. Furthermore, when the titania surface comprises many sites with highly reactive oxygen vacancies (V_O_), hydrogen molecules can be dissociative chemisorbed there with subsequent electron trapping at the corresponding unoccupied V_O_ in-gap states [84,85]. Finally, the use of CNTs as a dispersing template inhibits the clustering of oxide Nanoparticles (NPs) so increasing the efficiency of sensing devices based on Pt/TiO_2_ components. Furthermore, by changing the order in which CNTs are added to TiO_2_ solution, hetero-structures with different grain size and crystalline phase of the MOX were synthesized. Adding CNTs to the micro-emulsion solution favored the aggregation between oxide nanoparticles over the interaction with CNTs, leading to the formation of larger anatase titania agglomerates. Instead, smaller-sized rutile particles were formed by adding CNTs before the onset of precipitation. All these evidences are presented and discussed by Frontera et al. [86] that took care of the characterization of TiO_2_-CNTs/Pt nanohybrids, prepared by the micro-emulsion method, and used as active materials in electrochemical devices designed for amperometric sensing of hydrogen peroxide. It emerged that the nanocomposite consisting of uniformly distributed TiO_2_ particles on the surface of CNTs and smaller Pt nanoparticles showed the best sensing performance towards H_2_O_2_. The main result was that the TiO_2_-CNTs/Pt based sensor was able to work at lower potential (0.3 V). This is of obvious advantage, because it limits the interfering of other oxidizable species and provides a much wider linear dynamic range.

The lack of selectivity towards H_2,_ as well as sensor reliability in the presence of species other than H_2_ gas (leading to false alarms), constitute drawbacks of most conductometric sensors. For example, in car parking closed places where ventilation is insufficient, a CO high concentration is detected together with H_2_. Thus, sensors for the hydrogen detection need to be highly selective.

To enhance sensors sensitivity and selectivity, semiconducting MOX for hydrogen sensing are usually doped with suitable modifiers. The sensing characteristics of certain SnO_2_-based conductometric hydrogen sensors display substantial differences as a function of the concentration and working temperature, as reported in Ref. [87]. In order to better discuss this important aspect, the analytical performance of the developed sensor in comparison with other sensors reported in literature is summarized in Table 1. It can be seen that Co-SnO_2_ based conductometric sensors are characterized by a high response, especially with respect to those working at lower temperature. Furthermore, Co-doped SnO_2_ particles are characterized by catalytically active centers working effectively as H_2_ oxidation centers. The oxygen vacancies in SnO_2_ nanoparticles act as preferential adsorption sites for O_2_ coming from the gas phase. The more favorable surface reaction of the target gas with reactive adsorbed oxygen species determines the increase of gas response towards H_2_ [87]. Gas sensing measurements evidenced that the Co-doped SnO_2_ based sensor exhibited high sensitivity and good selectivity towards hydrogen compared to undoped SnO_2_ and Mn-doped SnO_2_. However, we outline that an effective improvement of the sensing characteristics occurs when Mn-doped SnO_2_ particles size decreases [87]. Comparing the response and recovery times, and also taking into account the concentration expressed in ppm and the sensors working temperature (see Table 1), we observe that the Co-doped SnO_2_ shows the highest response with respect to the other listed sensors, working in some cases also at lower temperature. By this comparison, the 10 wt% Co-SnO_2_ sensor has been indicated among the best hydrogen leak devices based on a conductometric platform, so it is the most promising candidate for highly sensitive and selective detection of H_2_ for automotive applications.

Nowadays, some drawbacks emerged using bulk Pd as sensing layer in H_2_ conductometric platforms. Thick Pd film can result in an extraordinary large internal stress leading to buckling of the films [88] which induces an irreversible resistance change. Moreover, at room temperature, hydrogen atom diffusion in Pd is very slow leading to a long response time [89]. Otherwise, Pd nanowires emerge as a promising alternative for the development of H_2_ sensors [90].

**Figure 8:**
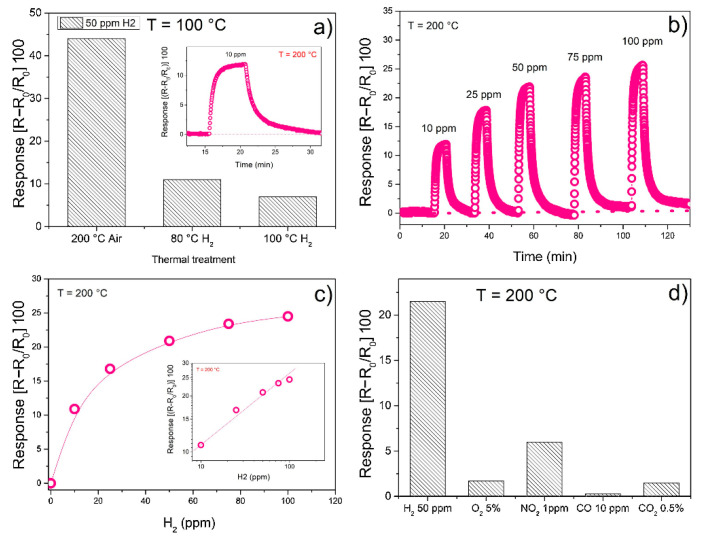
(**a**) Sensing response towards 50 ppm H_2_ for sensors working at 100 °C, but treated at the three different mentioned conditions; (**b**) Sensing response to pulses of hydrogen at concentration going from 10 ppm to 100 ppm, for the sensing film first annealed at 200 °C and working at 200 °C; (**c**) Calibration curve; (**d**) Sensing response to different gases at the indicated concentrations. Reprinted from Sensors and Actuators B: Chemical, Vol 262, Fazio E. et al., Synthesis, characterization and hydrogen sensing properties of nanosized colloidal rhodium oxides prepared by Pulsed Laser Ablation in water, Pages No. 79–85, Copyright (2018), with permission from Elsevier [37].

With the aim to explore the potential of other noble metal oxides as gas-sensing materials, Fazio et al. [37] prepared nanosized rhodium oxides (RhO_x_) by the green pulsed laser ablation technique. In a controlled temperature environment, the sensors’ resistance was measured by varying the hydrogen concentration. A chosen set of sensors was conditioned in air for about 2 h at 200 °C before sensing tests, while other two sensors were initially annealed at 200 °C in air then one of them was treated in a pure hydrogen atmosphere for 10 min at 80 °C while the other at 100 °C. As shown in Figure 8a, the highest sensor sensitivity was obtained working at 100 °C.

However, the sensor based on the film annealed in air at 200 °C was the most responsive, as indicated by the sensing response reported in Figure 8b. A completely reversible behavior was seen for low concentration of H_2_ (10 ppm), also for medium-high humidity levels. Moreover, this sensor shows a good stability after repeated cycling and the sensing response to 50 ppm of H_2_ was much higher than that to other simple gases tested including CO, CO_2_, NO_2_ and O_2_ (Figure 8d).

**Figure 9:**
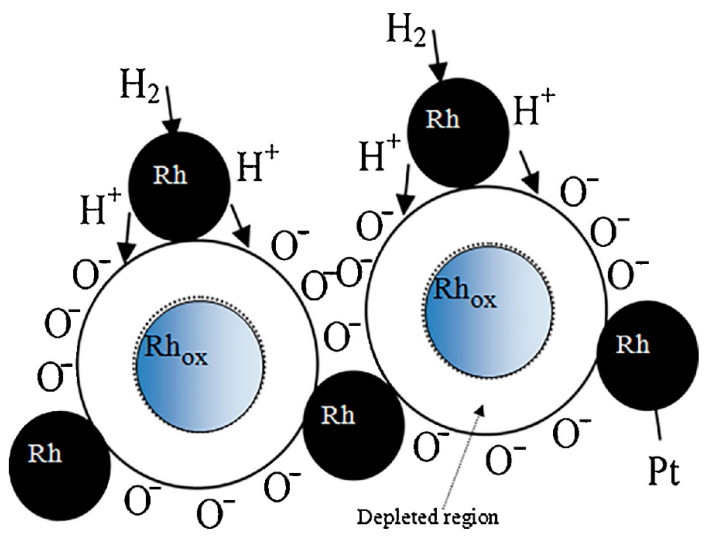
The working principle of hydrogen sensing mechanisms by considering the “spillover effect” over Rh/RhOx-based film. Some drops of the Rh-based colloidal solution was deposited by drop casting on alumina substrates supplied with interdigitated Pt electrodes. Reprinted from Sensors and Actuators B: Chemical, Vol 262, Fazio E. et al., Synthesis, characterization and hydrogen sensing properties of nanosized colloidal rhodium oxides prepared by Pulsed Laser Ablation in water, Pages No. 79–85, Copyright (2018), with permission from Elsevier [37].

Figure 9 reports a scheme for the sensing mechanism based on what is known as “spillover effect” over Rh/RhO_x_-based film [37].

**Figure 10:**
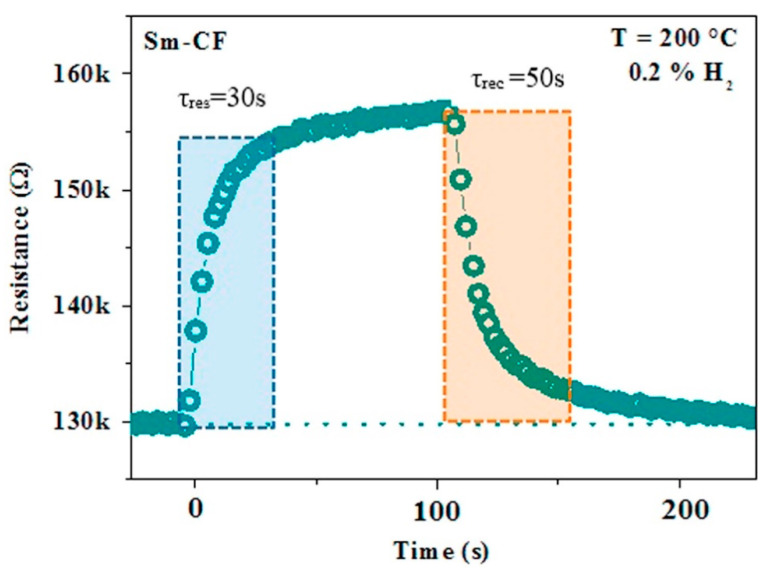
Transient response of the Sm-Cobalt ferrite (Sm-CF) sensor, showing the response/recovery time. Reprinted from Ceramics International, Vol 43, Falsafi F. et al., Sm-doped cobalt ferrite nanoparticles: A novel sensing material for conductometric hydrogen leak sensor, Pages No. 1029–1037, Copyright (2017), with permission from Elsevier [91].

Perovskite and spinel MOX systems are generally more stable under reducing atmosphere and then they are interesting for the monitoring of hydrogen. Sm-doped Cobalt ferrite (CoFe_2_O_4_) conductometric sensor exhibited good sensitivity, good reproducibility and stability over time and fast dynamics (see Figure 10 and Ref. [91] for more details). The Sm substitution into the cobalt-ferrite host structure offers a proper microstructure for H_2_ sensing. Less conventional MOX nanomaterials were also synthesized and processed for their application in conductometric sensors [92,93,94,95]. For example, another interesting activity about the MOX nanoparticles materials is that related to evaluating the effect of the irradiation by gamma rays (γ-rays) on their sensing properties [96,97]. WO_3_ NPs have been prepared by a microwave method and successively irradiated. The γ-irradiation with ^60^Co gamma rays at different doses (0, 50 and 100 kGy) induced significant changes on structural properties of WO_3_ nanoparticle and indeed in the MOX microstructure, favoring the tetragonal to triclinic transformation, along with a reduction of the grain size. The consequent effect on the sensing properties for detection of NH_3_, CO and CO_2_ in air was found to be dependent on the tested gas. The response to NH_3_ decreased after γ-irradiation, while that to CO_2_ increased. Further, γ-irradiated WO_3_ sensor displays fast recovery time for NH_3_, when compared to pristine WO_3_ sensor. Results reported for both WO_3_ and SnO_2_ demonstrated that the γ-irradiation can be an effective step for tailoring the sensing properties of MOX NPs.

## 4. Electrochemical Sensor: Building Basics and Sensing Mechanisms

Electrochemical sensors exploit the changes in the electrical signal due to bio-chemical reaction induced by a specific analyte at the working electrode surface [98]. Note that there are potentiometric sensors that do not exploit electrons but work on the principle of equal electrochemical potential [99]. In such cases, since the chemical potential of the species cannot be equal, compensation can be obtained through the generation of electrical potential at the phase boundary. Electrochemical sensors are particularly suitable for the determination of organic substances in liquid media, so they find interesting practical applications for the analysis of a variety of biomolecules in physiological fluids (blood, urine, saliva) and in other complex matrixes. For these applications, electrochemical sensors functionalized with enzymes and other biological receptors (biosensors), are generally used for their high selectivity towards the target biomolecules.

Nowadays, electrochemical sensors composed by MOX have received growing interest for the successful detection of electroactive biomolecules in many fields including medicine, environmental processes, energy efficient systems, food safety, chemical and agricultural industries. The demand for such metal oxide based biosensors continues to increase due to their ability in performing rapid measurements and analyses with flexible and reliable characteristics [12]. In fact, electrochemical sensors are able to convert, in a direct and fast way, biological events to an electronic signal with great stability. Furthermore, they can be combined in composite and flexible structures [100,101] and have high sensitivity and low-cost [102]. MOX based electrochemical sensors are also used to detect trace metals in the environment (especially in water). The determination of heavy metals is of vital importance in monitoring environment quality. Currently, a natural mechanism for controlled removal of heavy metals from the human body is unknown. Hence, even trace levels of toxic heavy metals, e.g., lead, cadmium, mercury and arsenic may have detrimental effects on the environment and human health. The standard approaches used to monitor trace of metals are atomic absorption/fluorescence and emission spectroscopies. Furthermore, potentiometric sensors are currently the most performing sensors since they cover a wide linear range (between 10^−7^ and 10^−1^ M), allowing the determination of metal ions in industrial wastewater where tens to hundreds of ppm of heavy metals are found [103].

However, the electrochemical sensors engineered today require time, manpower, expensive electrode materials that can only be used within a laboratory and chemical agents, and additionally suffer from multi-elemental interference. It is therefore clear that the above techniques are not realistic approaches for meeting the new EU regulations in a cost-effective manner. Instead, a cheap, fast and easy measurement protocol that can be performed in situ should be introduced for a widespread testing of heavy metals pollution. Particularly, the combination of electrochemical techniques (miniaturized and portable potentiostats adopting screen-printed electrodes) is considered a promising candidate to optimize determination methods, in terms of quality targets and to effectively monitor a wide variety of global health parameters that affect all of us [104].

**Figure 11:**
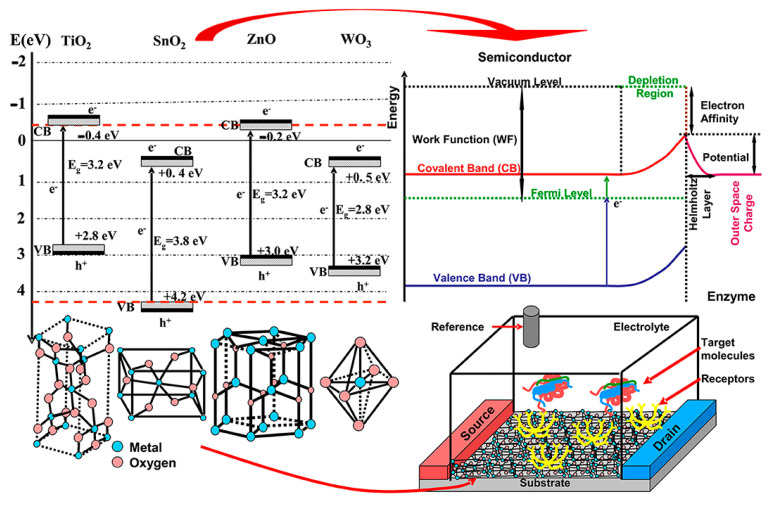
Working scheme of a Field Effect Transistor (FET) type of sensor based on MOX showing band energies, crystalline structure and biosensor configuration. Figure reused under the terms of CC-BY license from Ref. [12].

Figure 11 reports a schematic illustration about the structure and working principle of a Field Effect Transistor (FET) type of sensor based on MOX [12]. These sensors are mostly based on potentiometric principles and on the compensation of the generated potential at the gate by analyte binding on the gate surface [105]. The small time needed for charge accumulation on the nanomaterial channel between source and drain electrodes allows to quickly detect and analyze different analytes. Thanks to their enhanced sensitivity and selectivity, FET-based biosensors are very much employed in different fields [106]. Generally, biosensors can be considered as basically composed by two main elements: a biotransducer and the signal processing elements [107]. The biotransducer consists of a working electrode, a counter electrode and a reference electrode. The reference electrode, kept away from the reaction site, maintains a stable potential. The counter electrode, after interacting with the electrolytic solution, sends an electric signal to the working electrode, which is just the transduction element of the corresponding biochemical reaction process. Once the recognition of the target analyte happens, the probe molecules send signal impulses to the processing elements that can be indeed easily analyzed.

Generally, wide band gap semiconductors are used to construct such biosensors due to their unique crystalline structures and physical properties (electrochemical, optical, electronic, gravimetric and piezoelectric) [108]. The other advantages of MOX include their specific chemical composition, crystallization degree and that the interaction pathways between their surface and the analyte can be tuned to achieve a proper displacement of Fermi energy and induced depletion [109]. In fact, physico-chemical properties of the semiconductor surface are altered by the interactions with the analytes and corresponding changes can be correlated with the specific induced stimuli [110]. Furthermore, the biosensor surface can be functionalized to offer super hydro–phobicity/philicity, self-cleaning, antimicrobial activity and selective response to external stimuli such as light exposure [111].

From a general point of view, electrochemical responses corresponding to the reaction under investigation can be monitored by measuring potential, resistance and electric current. These can be divided into: (1) non-interfacial methods which are applied to the whole solution, such as conductometry [112] and (2) interfacial methods when the analyte is revealed on the electrode surface. The latter can be also divided into: (i) static if the electric current is null (e.g., potentiometry) and (ii) dynamic if an electron transfer (redox reaction) takes place between the electrode and the analyte (e.g., voltammetry and amperometry) [98,113].

Conductometric techniques are based on the measurement of conductivity (resistance) changes of an electrolyte solution because of a precise chemical reaction. Usually, they deal with enzymatic reactions that, by inducing changes of the ionic strength, and thus of the conductivity, provoke a quantifiable variation in the amount of the charged species in the considered solution [114]. Although their intrinsic low sensitivity usually limits their potential applications, the implementation of hybrid electrodes has opened new routes for their use in biosensing [115]. In addition, both the fast progress of semiconductor technology and the possible integration of sensors within microelectronic devices [116] brought a growing interest for biosensors using conductometric devices in combination with nanostructures [117].

Potentiometric techniques are based on the measurement of the potential corresponding to the electrical charges collected on the working electrode. Then, it is compared to that of another electrode (reference electrode) located inside an electrochemical cell, when negligible current flows through these two electrodes [118]. The detection limit depends indeed on the analyte, ranging from 10^−8^ to 10^−11^ M.

Voltammetric techniques are based on the measurement of the electric current flowing across the electrochemical cell as a function of the applied potential. Although the applied potential can be varied in different ways corresponding to different methods (e.g., cyclic voltammetry (CV), differential pulse voltammetry (DPV), square wave voltammetry (SWV)) [119], all these techniques involve the same quantities: potential, current and time. Although cyclic voltammetry is mostly a diagnostic and not an analytic tool, it is the most used voltammetric technique, allowing the measurement of the redox potential and rate of the chemical reactions which take place within the analyte solutions [98]. In details, the voltage, varied between two reference values at a specific scan rate, should be correctly chosen to provide enough time for the evolution of the chemical reaction. Hence, different scan rates furnish different results [118]. The electric current, measured between the working and the auxiliary electrodes, is then plotted as a function of the voltage applied between the reference and the working electrodes, producing the so-called voltammogram. Today, there is a growing interest in the development of biosensors also employing other kinds of electrochemical detection techniques, such as impedimetric that employs impedance measurements [120] and the field-effect which utilizes transistors for measuring the electric current after a potentiometric event at the gate electrode [115].

## 5. An Overview on MOX Nanomaterials Used for Biosensing Detection

In this section we report achievements, obtained by the authors in the last few years, and other relevant works reported in the literature on the implementation and optimization of MOX as biosensor components in biological and environmental systems. Mono and coupled semiconductors (composite, heterostructures etc.) are generally adopted. Among them, TiO_2_, MnO_2_, SnO_2_, MoOx, ZnO and WO_3_ metal oxides (also added with metal nanoparticles), carbon based materials or doped with metal ions are applied [12]. In order to optimize MOX nanostructures composition, morphology and structure, and in turn to enhance biosensing response, the synthesis parameters have been changed [121,122,123]. We focused on revealing neurotransmitters, which are endogenous chemical messengers playing an important role in many of the brain functions, abnormal levels being correlated with physical, psychotic and neurodegenerative diseases. Epinephrine (EP) is an excitatory neurotransmitter (NT) helping in regulating alertness, cognition, metabolism and mental focus, while Norepinephrine (NE) is another excitatory neurotransmitter vital for metabolism, heart rate and attention. Dopamine (DA, 3,4-dihydroxy phenylalanine), which belongs to the catecholamine family of neurotransmitters, consists of a benzene ring having two hydroxyl side groups with monoamine group attached via an ethyl chain. DA is mainly produced in adrenal glands and several areas of the brain, and it is also involved in brain-body integration. DA plays a significant role in the functioning of the central nervous, renal, hormonal and cardiovascular systems. Therefore, dopaminergic systems serve as a target for antipsychotic drugs and act as brain reward systems. Serotonin (5-hydroxytryptamine, 5-HT) is a redox active monoamine neurotransmitter, which is biochemically derived from tryptophan. 5-HT plays a crucial role in the emotional system by regulating mood, sleep, emesis, cardiovascular function and appetite. These classes of NTs have a great impact on the smooth running of the central nervous system. Hence, it is very important to precisely quantify NTs in extracellular fluid for an easy diagnosis of health conditions associated with the imbalance in the level of any or all of them in the human body system [124]. In this case, electrochemical sensors, being characterized by high sensitivity, wide linear range, fast response time and low limit of detection, can be efficiently used for precisely monitoring NTs [125,126,127].

**Figure 12:**
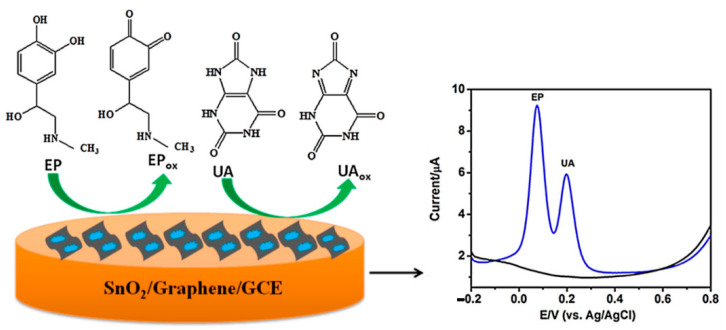
Schematic representation of the SnO_2_/graphene modified glassy carbon electrode for simultaneous detection of epinephrine (EP) and uric acid (UA). Reprinted from Sensors and Actuators B: Chemical, Vol 221, Lavanya N. et al., Simultaneous electrochemical determination of epinephrine and uric acid in the presence of ascorbic acid (AA) using SnO_2_/graphene nanocomposite modified glassy carbon electrode, Pages No. 1412–1422, Copyright (2015), with permission from Elsevier [128].

Simultaneous detection of epinephrine (EP) and uric acid (UA) in the presence of common interferent ascorbic acid (AA), in different human fluids such as plasma and urine, has been reported by Lavanya et al. [128]. This is of great interest for investigating their physiological functions and diagnosing diseases. However, EP, UA and AA are oxidized at almost similar potentials with poor sensitivity at bare solid electrodes and the overlap of their voltammetric responses would confuse their simultaneous determination. Interestingly, SnO_2_/graphene composite modified glassy carbon electrode (GCE) enabled simultaneous determination of EP and UA in the presence of AA with good separation in the oxidation potential. The determination of EP and UA was possible by cyclic voltammetry method using a SnO_2_/graphene composite modified glass electrode with Ag/AgCl standard electrode. The application of the specific redox potential allows the oxidation of alcoholic to ketonic groups, producing a quinone functionality in the EP molecule. Otherwise, the amine oxidation to imine groups is observed on the UA molecule. The developed sensor (see Figure 12) showed better electrochemical performance for the oxidation of EP and UA compared to the bare GCE and SnO_2_/GCE, possibly due to the high surface area and synergistic effect of the composite materials. Moreover, the SnO_2_/graphene/GCE showed a simple, rapid and sensitive protocol for the simultaneous determinations of EP and UA with the lowest detection limits of 0.017 μM and 0.28 μM [128], with respect to other different chemically modified electrodes (see Table 2).

These promising results have stimulated the research activities about another GCE modified electrode, namely Mn doped SnO_2_ nanoparticles modified electrode (Mn-SnO_2_/GCE). The Mn-SnO_2_/GCE has shown wider linear range and low detection limits for the simultaneous determination of AA, UA and folic acid (FA). The linear responses of AA, UA and FA were tested in the concentration ranges of 1 to 900, 1 to 860 and 0.5 to 900 μM for AA, UA and FA, with detection limits of 56, 36 and 79 nM respectively. For simultaneous determination by synchronous change of the analyte concentrations, the linear response ranges were between 5 and 500 μM for UA and 1–500 μM for FA, with the lowest detection limits of 25 and 38 nM respectively, in the presence of AA [129].

Swamy et al. [126] reported cyclic and differential pulse voltammetry studies using metal oxides (Cu, Ni) aimed at simultaneous determination of AA, DA and Tyr. CV studies with Tyrosine (Tyr) at MO modified electrode (M = Cu, Ni), showed an irreversible oxidation process and both modified electrodes exhibited an anodic peak at a potential of +0.80 V, against very low or no anodic peak currents obtained at bare graphite electrode. Moreover, the CuO modified electrode successfully separated the anodic signals of dopamine (DA), ascorbic acid (AA) and Tyr in their ternary mixture whereas, on bare graphite, a single, overlapped oxidative peak was observed. In CV studies, the peak potential difference between AA-DA, DA-Tyr and AA-Tyr is 166 mV, 323 mV and 489 mV respectively and the corresponding peak potential separations are 209 mV, 400 mV and 609 mV respectively in differential pulse voltammetry (DPV). On the other hand, NiO modified electrodes display poor activity towards DA, but show good sensitivity towards the determination of Tyr, while CuO modified electrodes show remarkable sensing activity towards multianalyte mixture of DA, AA and Tyr.

**Figure 13:**
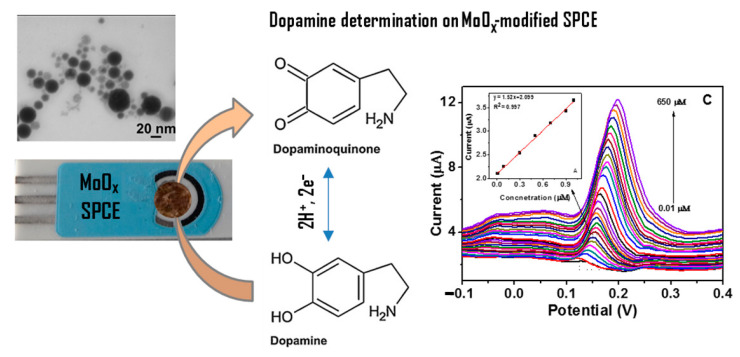
Schematic representation of dopamine electrochemical oxidation on the MoO_x_ nanostructured modified screen-printed carbon electrode and square wave voltammetry (SWVs) for 0.01–650 μM dopamine (DA) concentrations at MoOx/screen printed carbon electrode (SPCE) in 0.1 M phosphate buffer saline solution. The inset shows the calibration curve at low DA concentration (0.01–1 μM). Reprinted from Journal of Electroanalytical Chemistry, Vol 814, Fazio, E. et al., Molybdenum oxide nanoparticles for the sensitive and selective detection of dopamine, Pages No. 91–96, Copyright (2018), with permission from Elsevier [130].

The combination of the efficient electron redox capability of pulse laser ablation (PLA) synthesized MoOx NPs colloids with the fast electron transfer rate of screen printed carbon electrode (SPCE) emerged as a good possibility for obtaining sensitive and selective detection of DA, excluding any interference from ascorbic acid [130]. This is explained on considering the high surface to volume ratio and Mo participation to surface oxidation processes. Figure 13 reports a schematic picture of the electrochemical oxidation of DA using MoOx NPs/SPCE structure. In this case, the DA content is detected without any electron transfer mediator (such as graphene, carbon nanotube, etc.).

In addition, MoO_x_NPs/SPCE sensing performance (linear range: 0.01–650 μM, limit of detection (LOD): 43 nM) is comparable to that shown by a ternary composite including reduced graphene oxide(rGO) that is MoO_2_-rGO/polyimide (linear range: 0.1–2000 μM, LOD: 21 nM) [131]. Finally, the preparation procedure is very complex for the ternary composite with respect to the samples synthesized by the picoseconds pulsed laser ablation (ps-PLA) which is a very simple, green and cheap method [131,132].

Regarding serotonin (SE) detection, MnO_2_ nanoparticles have been anchored on graphene (GR) support, yielding MnO_2_-GR composite with a large surface area, improved electron transport, high conductivity and numerous channels for rapid diffusion of electrolyte ions [133]. Indeed, even if MnO_2_ is one of the most promising transition MOX for electrochemical applications due to its non-toxicity, environmental compatibility and low cost [134,135], it is characterized by relatively poor electrical conductivity.

**Figure 14:**
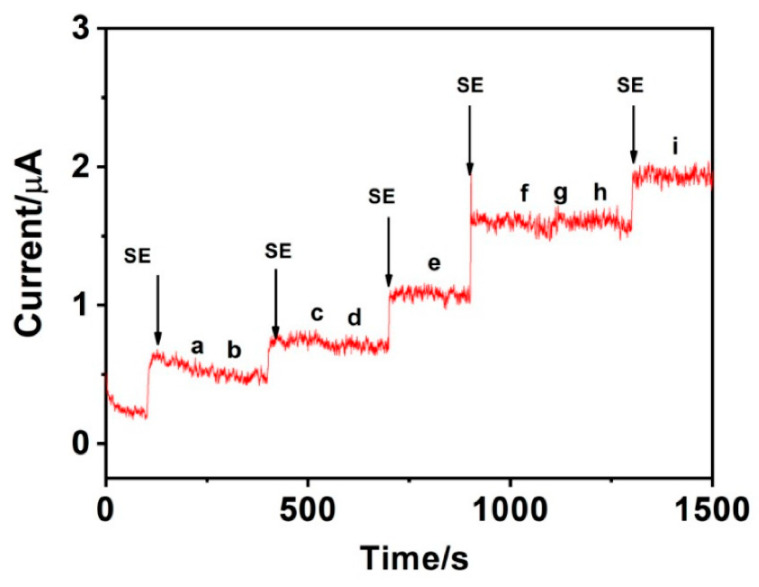
Amperometric response of MnO_2_-graphene (GR) modified glassy carbon electrode (GCE) for the addition of 50 µM serotonin (SE) and successive addition of interferents 500 µM each in the sequence of (a) DA, (b) EP (c) folic acid (FA), (d) UA, (e) AA, (f) glucose (g) Na^+^, (h) K^+^ and (i) Mg^2+^ in 0.1 M PBS buffer. Figure reused under the terms of CC-BY license from Ref. [133].

Recently, Lavanya et al. [133] synthesized a MnO_2_-GR composite by the microwave irradiation method and fabricated an electrochemical sensor for detection of serotonin (SE) (see Figure 14). Microwave heating increases the rate of certain chemical reactions by several folds when compared to conventional heating. In addition, it does not produce any green gas or other side products and the use of solvents in the chemical reaction can also be removed or reduced significantly. The developed sensor showed an excellent electrochemical activity towards the detection of SE in phosphate buffer saline (PBS) at physiological pH of 7.0. Tests were made by carrying out square wave voltammetry (SWV) measurements, over a wide linear range of 0.1 to 800 µM, with the lowest detection limit of 10 nM (S/N = 3), with a good anti-interference ability, high reproducibility and long-term stability. It has been found that 100-fold concentrations of Na^2+^, K^+^ and Mg^2+^ and 10-fold excess DA, EP, FA, UA, AA and glucose (500 µM) had no obvious influences on the response of 50 µM serotonin with deviations below ±5%. Moreover, the oxidation potential (0.6 V) of nor-epinephrine (NE) is far from that of SE (0.4 V), therefore the interferent NE will not influence SE detection.

**Figure 15:**
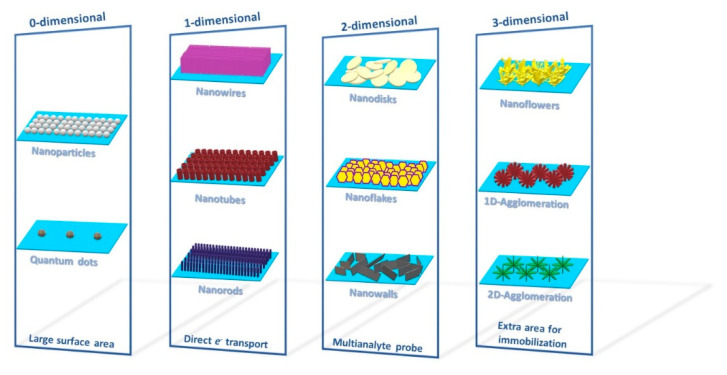
Four different dimensions of ZnO nanostructures with their advantages. 0D nanostructures provide large surface area. 1D nanostructures possess stable and direct electron transport. 2D nanostructures give specific planes for immobilization process for the simultaneous detection of different analytes. 3D nanostructures have extra surface area (outer and inner area) to provide more sites for immobilization.

Nowadays, biosensors based on ZnO nanostructures are largely used for detection of multiple analytes. Different ZnO nanostructures and their advantages in terms of sensing applications are shown in Figure 15. As described in Ref. [136], zero dimensional (0D) nanostructures provide large surface area, one dimensional (1D) nanostructures possess stable and direct electron transport, two dimensional (2D) nanostructures give specific planes for immobilization process for the simultaneous detection of different analytes and finally three dimensional (3D) nanostructures have extra surface area (outer and inner area), to provide more sites for immobilization. For example, nanohybrid ZnO and reduced graphene oxide have been used for AA and DA sensing [137], while 0D ZnO nanoparticles with sizes in the range of 10–100 nm have been successfully adopted to fabricate miniaturized medical biosensors [138]. ZnO nanomaterials are particularly suited for glucose detection, whose released electrons provoke an extension of the depletion layer and a decrease in the electric current proportional to the number of glucose molecules [136]. On the other hand, 1D ZnO nanostructures (nanorods, nanotubes, nanofibers and nanowires), showing an increased surface/volume ratio with respect to 0D nanostructures, provided a direct pathway for fast electrons transport and then have been successfully implemented for efficient glucose sensing with a sensitivity of 10.911 mA/(mM cm^2^) and a lower detection limit of 0.22 mM [139]. Furthermore, functionalization of ZnO nanotubes with molecularly imprinted polymer (MIP) allows reducing selectivity problems among different analytes [140]. As previously mentioned, 2D ZnO nanostructures (nanosheets, porous nanoflakes, nanodiscs and nanowalls) allow an optimal immobilization of enzymes. At the same time, ZnO nanosheets have been proven to offer a bio-compatible surface able to retain the cytochrome-c bioactivity and to sustain its natural activity towards H_2_O_2_ [141]. Moreover, ZnO nanowalls with stabilized polymerized films have been used to detect cholesterol [142]. In such a case, the geometrical properties of the nanowalls together with the cholesterol solubility exhibited by the lipid matrixes have shown a significative cholesterol oxidase absorption. ZnO nanowalls have the unique possibility to alternate positive and negative layers along their nonpolar planes, so facilitating the cholesterol oxidase absorption.

As already outlined, the precise quantification of glucose and DA is crucial both for analytical applications and in diagnostic research, since they are key in physiology and above all are coupled with important diseases such as diabetes mellitus, Parkinson’s disease and schizophrenia [143,144]. It is well known that the amount of DA, being a fundamental catecholamine neurotransmitter in the mammalian central nervous system, affects the body physiological functions [145], and it is also correlated with brain glucose metabolism that, in neuronal activity, triggers the transients of vesicular neurotransmitter release and fluctuations of metabolites in the proximity of the activated neurons [146]. Recently, a simultaneous detection of glucose and DA was carried out using CuO and hybrid nanostructures composed by CuO and graphitic carbon nitrides (g-C_3_N_4_) [147,148,149,150]. Notably, g-C_3_N_4_ is an innovative two-dimensional π-conjugated material containing many nitrogen atoms and defects that, by generating delocalized electrons, allow high metal coordination sites as catalytically active sites [143,151]. The mechanism of glucose oxidation on g-C_3_N_4_/CuO in NaOH can be explained by the following equations [143,151]:

CuO + OH^−^ → CuOOH + e^−^

CuOOH + e^−^ + glucose → CuO + OH^−^ + gluconic acid

Ultimately, CuO based biosensors, from one side, can achieve the direct electrocatalytic oxidation of glucose and, from the other side, can detect DA being an electroactive compound [143,152,153].

The electrochemical behavior of different types of CNTs and the effects of their different orientation, size, morphology and oxidation treatments towards the oxidation of H_2_O_2_ have been widely investigated in the last years [154]. Decoration of CNTs with Pt NPs has been proposed [155] to improve the electrocatalytic properties towards H_2_O_2_ monitoring. The presence of metal improves the electrochemical activity, reducing the oxidation overpotential compared to platinum-free carbon nanostructures. In particular, the presence of small and well-dispersed Pt nanoparticles plays a key role in promoting the electrocatalytic activity towards H_2_O_2_ oxidation. In details, the occurrence of a low Pt^0^/Pt^2+^ ratio seems to favor the adsorption of H_2_O_2_ and its discharge, contributing to enhance the electrocatalytic activity, exhibiting a high sensitivity (177 µA mM^−1^ cm^−2^) [156]. Moreover, TiO_2_-CNTs/Pt nanohybrids were used as active materials in electrochemical devices designed for amperometric sensing of H_2_O_2_. When smaller titania and Pt particles were obtained, better electrochemical properties were registered, while sensing towards H_2_ in gas phase was little influenced by this [86]. The selectivity of this sensor was also examined recording the responses towards some of the most common substances, that are present in biological and environmental samples, and which could cause interferences during electrochemical determination of the target analyte [157]. A clear and fast increase of the current is observed when 0.1 mM of H_2_O_2_ is added, no significant response is observed instead for 0.1 mM of NaCl, KCl, KNO_3_ and CaCl_2_ salts, as well as for the same concentration of citric acid (CA). As regards AA, DA and UA, no significant interference is observed for concentrations up to 5 µM.

**Figure 16:**
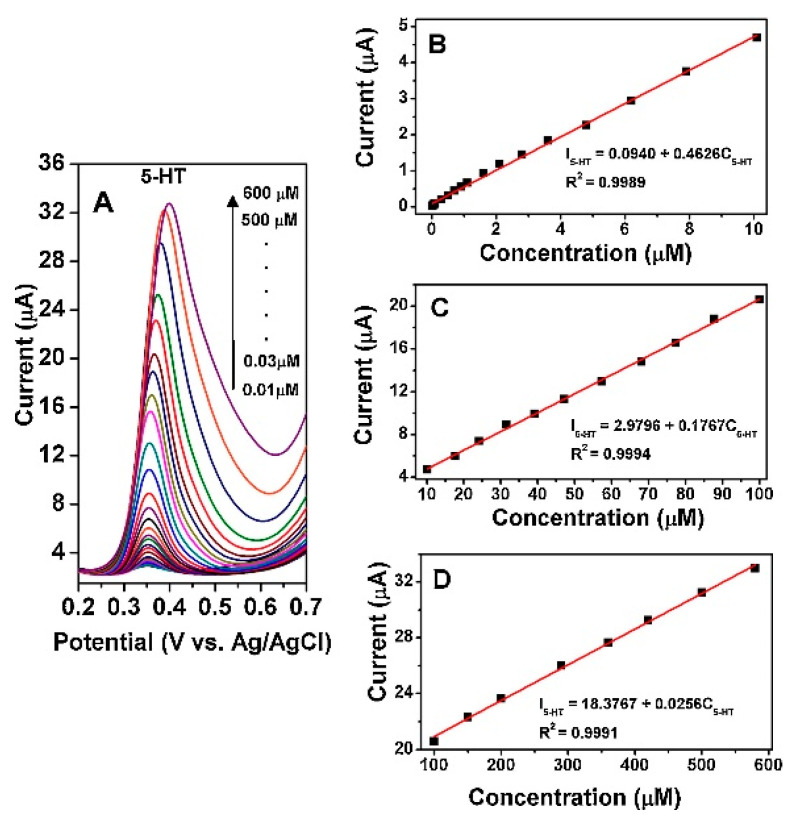
(**A**) differential pulse voltammetry (DPV) of 100 kGy WO_3_/GCE in 0.1 M phosphate buffer saline (PBS) (pH 7.0) containing different concentrations of serotonin (5-HT) (0.01–600 µM). (**B**–**D**) show the plots of the electrocatalytic oxidation peak current as a function of 5-HT concentration within the range of 0.01–10 µM, 10–100 µM and 100–600 µM, respectively. Reprinted from Sensors and Actuators B: Chemical, Vol 238 Anithaa A.C. et al., Highly sensitive and selective serotonin sensor based on gamma ray irradiated tungsten trioxide nanoparticles, Pages No. 667–675, Copyright (2017), with permission from Elsevier [158].

Among other metal oxides, tungsten trioxide (WO_3_), exhibits fascinating electronic, structural and mechanical properties with a wide range of applications in the areas of gas sensors, electrochromic, photochromic and electrocatalytic processes. It is an intrinsically n-type semiconductor, the stoichiometric excess of metal being due to oxygen vacancies. Anithaa et al. have synthesized WO_3_ nanoparticles by microwave irradiation method and subsequently modified the surfaces through gamma irradiation under different doses (0–150 kGy). Differential pulse voltammetry (DPV) studies carried out at 100 kGy irradiated WO_3_ modified GCE in the presence of serotonin (SE) exhibited strong oxidation peaks (Figure 16) over a very wide concentration range of 0.01 µM to 600 µM SE in 0.1 M PBS (pH 7.0). The fabricated sensor showed high sensitivity with the LOD of 1.42 nM with the signal to noise ratio (S/N) of 3, long term stability, excellent reproducibility and high selectivity towards potentially interfering substances [158].

The same authors demonstrated that the irradiation of WO_3_ with low energy nitrogen ion beam (fluences: 1 × 10^14^ to 1 × 10^17^ ions/cm^2^) and swift heavy ion [Ni^11+^] enabled the detection of acetylcholine [159] and guanine [160] with high precision and improved selectivity. In all the cases, WO_3_ NPs surface modification through irradiation seems to enhance the sensitivity, linear range and selectivity.

## 6. MOX-Based Sensors Drawbacks and Future Perspectives and Challenges

Data reported in this review are an example about the recent and effective applications of electrical and electrochemical sensors based on multi-component nanomaterials (of various shapes, sizes, chemical compositions and surface functional groups). Nevertheless, it has been remarked that the repeatability and reproducibility of the analytical information provided by these sensors can be influenced by the complexity of multicomponent materials. Therefore, specific requirements with regards to the intended application must be considered. The main drawbacks and the potential approaches to overcome them are summarized here:

(i) Low selectivity and low response/recovery speed for a long time and after repeated bending/recovering, without degradation of the sensor components. In this respect, one should take advantage of the light illumination of conductometric sensors to improve their sensing response at room-temperature operation.
(ii) Restricted sensing performance at room temperature, also due to the influence of humidity level. Thus, NRT gas sensors with a rapid response should be still engineered to meet the need for timely triggering of the alarm.
(iii) High degree of responsivity and selectivity for multiple-agent sensors should be still reached.
(iv) The interaction between the target molecules and chemisorbed oxygen species (such as O^2−^ and O^−^ ions) is almost known, a clear understanding of the interaction mechanisms of some groups bearing oxygen atoms (such as OH^−^) with the target molecules is missing. This investigation could be the starting point to develop surface modification procedures useful to minimize OH^−^ effects. As regarding biosensors, the peculiar chemical-physical properties that metal oxide nanohybrids on appropriately modified electrodes offer (with respect to other materials conventionally used to fabricate these biosensors) have been described in this review in view of specific sensing applications.
(v) A limited production of flexible and wearable sensor arrays for electroactive biomolecules detection; this is due to the relatively low mechanical robustness (mainly on flexible substrates) currently obtained. Therefore, this is still the major challenge to be addressed in gas sensors manufacture.

**Figure 17:**
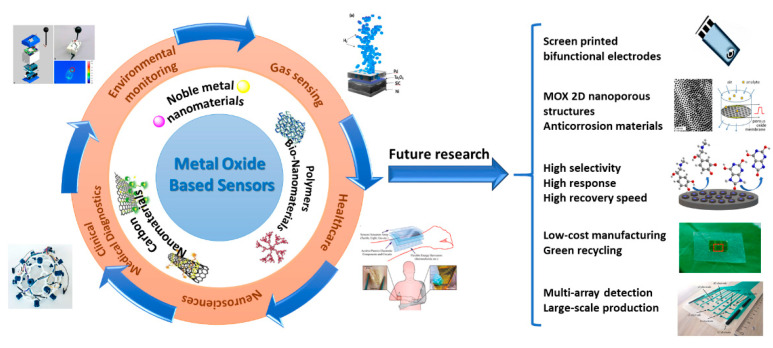
Illustration about the main recent applications and future research directions of sensors. Figures are reused under the terms of CC-BY license from Refs. [161,162,163,164,165,166,167].

In future, all these limits must be overcome by engineering MOX nanomaterials for a more universal use of the sensors. In addition, future research directions should bridge the gap between new electrochemical sensing concepts and real-world analytical applications. To convey the idea, a non-enzymatic glucose sensor showing excellent sensitivity to glucose in 0.1 M NaOH may be not ideal for wearable glucose monitoring under physiological conditions. This makes easy to understand as various analytical measurement scenarios will occur in the future using electrochemical sensors to their full potential. Figure 17 illustrates the main recent applications and future research directions of sensors.

Finally, further research and development is necessary to allow the commercialization of implantable in vivo and portable in vitro biosensor-devices, which require the improvement of practical, affordable and advanced nanomaterial-based electrocatalysts with multifunctional reactivity. In this context, electrochemical sensing parameters of advanced nanomaterials with bifunctional electrodes should be analyzed in future to understand the mechanism for the electro-catalytic activity of nanomaterials (mainly 2D materials). For example, all that will improve prospects for meeting the urgent need for point of care (POC) devices and live cell monitoring through low-cost miniaturized potentiostats.

## 7. Conclusions

In this review, MOX semiconductor-based electrical and electrochemical sensors used for gas sensing and for the determination of electroactive biomolecules are described in terms of their sensing performance and, in some cases, of their practical limitations to be used for multi-detections of gases and analytes.

As regarding conductometric gas sensors, a lot of literature has been produced through the years on sensing materials outlining the actions taken to optimize materials chemical-physical properties by a fine check of preparation/doping procedures. Here, more attention is instead focused on highly sensitive electrical and electrochemical sensors based on doped-SnO_2_, RhO, ZnO-Ca and Sm_x_-CoFe_2−x_O_4_, proposed to detect toxic and hazardous gases (H_2_, CO, NO_2_) and volatile organic compounds (VOCs) (e.g., acetone, ethanol). They have been applied in relevant applications such as monitoring gaseous markers in the breath of patients with specific pathologies, for the control of environmental pollution, home and industrial safety. In this review, we remarked the existence of a large variety of conductometric gas sensors based on MOX nanostructured materials, outlining that none of them can be considered an ideal gas-sensitive material. Each of them shows advantages and disadvantages: some have low selectivity, others increased sensitivity to humidity, some are stable only at low temperatures and some require high temperatures for efficient operation. Therefore, when choosing a MOX-based gas sensing material, it is necessary to take into account the type of sensor being developed, the nature of the gas, the sensor manufacture and operating conditions.

In the field of electrochemical sensors, the growing of advanced nanomaterials may support the next generation of new sensor devices for the biomedical and environmental field. As advantages, the design of the nanometer MOX characteristic factors such as shape, size, architecture, composition and functionalization may offer exceptional electrocatalytic properties, for improving the sensitivity and stability of the electrochemical sensor platform. However, besides these advantages, there are numerous characteristic drawbacks to be taken into account while designing the electrode materials. Indeed, advanced nanomaterials used in electrochemical sensors are required to offer high specificity and selectivity towards the target analyte. Thus, it is important in the design of the nanoscale electrode materials, not only to focus on the signal intensity but also to provide the right chemical interaction with the target biomolecule, which is prominent to highly selective sensing. Further, the optimization of electrochemical sensing parameters (e.g., electrode potential) will help in this, especially in the multi-analyte sensing.

In conclusion, in this review we tried to provide future research directions by specifying the many advantages but also highlighting the existing hindrances. Thus, the reader can critically acquire some ideas for the development of high performance electrical and electrochemical sensors based on the peculiar properties of MOX nanomaterials.

## Figures and Tables

**Table 1:** Comparison of the sensing characteristics of SnO_2_-based conductometric hydrogen sensors. Reprinted from International Journal of Hydrogen Energy, Vol 42, Lavanya N. et al., Development of a selective hydrogen leak sensor based on chemically doped SnO_2_ for automotive applications, Pages No. 10645–10655, Copyright (2017), with permission from Elsevier [87].

Materials	Working Temperature (°C)	Concentration (ppm)	Response (Ra/Rg)	Response/Recovery Time (s)
SnO_2_ nanowires	150, 300	1000	6.5, 4.25	-/-
Co-SnO_2_ nanofibers	330	100 (1000)	24 (~90)	2/3 (-/-)
SnO_2_ nanowires	300	1000	4.25	-/-
SnO_2_ thin film	r.t.	1000	26.5	192/95
Pt/SnO_2_ thin film	110	500	169	6/57
Pd-SnO_2_/MoS_2_ composite	r.t.	5000	1.22	30/20
Pd-SnO_2_ thin film	180	500	6.5	-/-
Pd-SnO_2_ nanofibers	280	100 (1000)	8.2 (~26)	9/9 (-/-)
Al-SnO_2_ nanofibers	340	100 (1000)	7.7 (~15)	3/2 (-/-)
ZnO/SnO_2_ composite	150	10,000	10	60/75
SnO_2_/CNTs	100	1000	1.55	-/-
Au-SnO_2_ NPs	250	100 (1000)	25 (150)	1/3 (-/-)
Eu-SnO_2_ NPs	350	300	21	7/-
RGO-SnO_2_ nanofibers	60	1000	1.3	119/265
Co-SnO_2_ NPs	250	2000	100	3/15

**Table 2:** Comparison of different chemically modified electrodes for EP and UA determination using SnO_2_/graphene/GCE. Reprinted from Sensors and Actuators B: Chemical, Vol 221, Lavanya N. et al., Simultaneous electrochemical determination of epinephrine and uric acid in the presence of AA using SnO_2_/graphene nanocomposite modified glassy carbon electrode, Pages No. 1412–1422, Copyright (2015), with permission from Elsevier [128].

Electrode	Linear Range (μM)		Detection Limit (μM)	
	**EP**	**UA**	**EP**	**UA**
Nano-diamond/graphite/PGE	0.01–10	0.01–60	0.003	0.003
Nanofion-OMC/GCE	0.5–200	0.25–100	0.2	0.07
Poly(*p*-xylenolsulfo-nephthalein)/GCE	2–390	0.1–560	0.1	0.08
Electrochemically activated GCE	1–40	1–55	0.089	0.16
Caffeic acid/GCE	2–80	5–300	20	60
CNTs/Ru oxide/hexacyanoferrate/GCE	0.1–10	0.90–250	0.087	0.052
Graphene/SnO_2_/Au composite/GCE	0.5–100	2–100	0.050	0.5
SnO_2_/graphene/GCE	0.5–200	0.1–200	0.017	0.28

## Data Availability

No new data were created or analyzed in this study. Data sharing is not applicable to this article.
